# Institutional Amenities

**Published:** 1898-11-05

**Authors:** 


### Editor's Letter-Box.

[Our Correspondents are reminded that prolixity is a great bar to publication, and that brevity of style and conciseness of statement greatly facilitate early insertion.]

### INSTITUTIONAL AMENITIES.

Sir,—It may interest the readers of the clever article on "Institutional Amenities" which appears in your this week's issue to hear the following little anecdote. Some time ago, early this year, I received a letter from a journal I will style the *Friday Review*, whose editor was said to be about to write a "series of articles" on "Hospitals and Their Modes of Working"; the editor wished me to meet his representative, who would call upon me if I would appoint a day and hour. The *Friday Review* is an old and well-known journal, and I was aware that in meeting a representative of such a leading organ I must exert my utmost ability to afford him all the desired information; my sense of responsibility was by no means lessened when the representative arrived, for he was not slow to inform me that he was regarded as an "expert at hospital work." You can imagine that having received him with my best, shall we say, "Chicago manners," and comfortably seated the gentleman in the best chair my office can provide, I was especially delighted when he informed me that the *Friday Review* meant to make special favourable mention of my institution in the coming series of articles. It struck me as more than kind that he should announce his intention to do this before even seeing our humble little hospital, or before he had been afforded an opportunity of knowing anything of its needs or merits. He next roundly and soundly slated the Prince of Wales' Fund, and said that the "article" would take it to task along with the Hospital Sunday and Saturday Funds for the unfair manner in which it distributed the moneys at its disposal. My heart glowed at the thought that at some future date, by means of this gentleman's caustic pen, I should find myself signing a receipt for a cheque tenfold as handsome as the last we received from the Prince's Fund. At the same time I could not help feeling that his ideas as to the methods of distribution followed by the public bodies he named were so strangely deficient that he was in no way qualified to criticise them in this respect. Then at the expert's initiation we went over the wards, and I showed him our fifty children's cots of which we are all so justly proud. The matron and her staff were there, spotless, superb, and ready for the eye of the most scathing critic. Strange as it seemed, there was still something odd about this expert, and as he turned down the sheets of a cot and carefully examined it with the air of a connoisseur, or cast a knowing eye at the formidable-looking electric appliances upon the wall, it occurred to me that his knowledge of hospital affairs was less extensive than might have been expected of a representative and an expert from the *Friday Review*. It was curious, too, that when speaking of finance he did not know the difference between maintenance and administration, and far from being versed in the institutional topics of the day, the out-patient question, &c., the usual phraseology of hospital life seemed Greek to him. I lost over an hour of my valuable time talking to this man, and he left me doubtful yet expectant of a good leg up from the *Friday's* series of articles. Some weeks later I received a letter from Mr. ——, of the advertisement department of the *Friday Review* asking for an order for an advertisement, the cost of which would have added 40 per cent. to our official advertisements. However, being of an inquiring turn of mind that morning, I was not long before I discovered Mr. —— had precisely the same hand-writing as the gentleman who had written the original letter announcing the series of articles on "Hospitals and their Modes of Working," and a further investigation showed that the writer of the original letter, my expert visitor, and the business gentleman who wanted to add 40 per cent. to our official advertisements were one and the same person. If journals of the reputation of the *Friday Review* let advertisement touts visit the hospitals in the disguise of Pressmen, can it be wondered that the more "gouty" secretaries are a little suspicious of some of the members of the profession? I don't know whether the articles in question ever appeared; I do not care. But this I do know, that the office battle-axe has lately been repolished, and as I write it is by my side ready for use upon the head of the next representative of the *Friday Review* who shall chance to favour me with a call.—Your obedient servant,

A Hospital Secretary and a Pressman.

